# Christian Science

**Published:** 1888-10

**Authors:** 


### HALL'S
### JOURNAL OF HEALTH

TRUTH DEMANDS NO SACRIFICE; ERROR CAN MAKE NONE.

VOL. 35. OCTOBER, 1888. No 10.

### CHRISTIAN SCIENCE.

Christian science, as it is termed in its relation to the cure of disease, is only another name for the "faith cure," which has had a rapid and wide-spread run, and is as yet, far from being run out. All those systems which are made to rely wholly upon the action of the mind of the patient when brought under the influence of other minds, are either based upon the supposed special interposition of Providence, or the theory that disease is the result of a disordered imagination, and has no existence in fact.

On the one hand, the endeavor is to induce by prayer and supplication, the all powerful aid of Deity to effect the cure; on the other, there is a recognition of the supremacy of mind over all forms of matter, and particularly, the individual mind over the anatomical structure through whose organs it expresses itself.

Physiologists compare the human organism to an electrical machine with its connecting wires: The brain answers to the battery; the sensatory nerves to the transmitting wires, with their relays of cells, and the will, to the operator whose touch sends the current upon its travels. The millions of cells of which the body is composed are constantly undergoing waste and replenishment. Every effort of the mind or body is at the expense of celular decay, and the means must be at hand for their resupply by purely natural processes, or the health of the body becomes permanently impaired. Such indeed appears to be the order of nature throughout its wide domain, out of death comes life. When the power of recuperation is lost in the general decay which overtakes all created things in their periods of decline, the change which we call death, ensues.

These natural processes, by means of which alone, the worn out tissues are ejected and new ones supplied and the impaired body restored to healthfulness, are wholly ignored by the Christian scientists and faith curists, who ascribe the healing of diseases under their methods, to the miraculous interposition of the Almighty. If we are to believe the statements of many who claim to have been restored to health through the prayerful supplication of the practitioners of Christian science, or their unquestioning faith in supernatural agencies in their behalf, there would seem to be abundant facts to sustain these most extravagent pretentions.

There can, indeed, be no doubt that many persons through weakness and long suffering, give themselves up to die without the energy to make the least mental or physical effort toward restoration, even though no real disease is apparent. With such, it is the mind alone that requires reassuring, and a strong counter action in the right direction will often effect a change from hypochondria to convalescense and eventual health. For such, the prayer is perhaps the needful remedy, and faith one of its essentials.

At our present writing that terrible scourge of the Southern States is running its deadly course, and some of the fairest of their cities are almost depopulated, save by the helpless sick and a few heroic physicians and nurses, who have charitably gone to their relief. But in these infested localities where there is so much suffering we hear nothing of the faith cure, or restoration by any of the means adopted by its adherents. Of all systems relating to the cure of disease, this, with its concomitants, including the prayer cure, is the only one that may be called religious, hence, we naturally enough look to it for a wider expression benevolence toward the afflicted, than to any of the drug dosing systems. Does this seemingly indifference imply that yellow fever and other contagious diseases are so far beyond the limits of the faith cure that neither faith nor prayer are able to produce any effect upon them, and are the efforts of their votaries to be confined to indolent hypochondriacs and bedridden sufferers whose ailments have been petted and nursed until they have mastered their will and become *per se*, the sole objects of their solitude? We do not deny the efficacy of the faith cure in such cases, nor that it has been ascribed to the right source, since all power is of the spirit.

But the immediate cause of the cures which have been effected at harmonious gatherings, wherein a singleness of purpose is the governing rule and all minds are centered upon the same object, has never yet been understood, much less appreciated by the practitioners of this, the very oldest system of healing; revived in the present. Its true solution is to be found in the spiritual philosophy, which, strange to say, has been, with great unanimity, resisted by the various church organizations, and condemned as a devil's creed in the face of overwhelming proofs of its actuality, and the solemn fact that its doctrinal truths lie at the base of all religions.

This philosophy not only teaches, but by following out its teachings, we are able to demonstrate, that every human being is incompassed in an aura or atmosphere which emanating from himself, being so to speak, formed of his own characteristics, is very surely determinative of his moral and intellectual value. Many persons of delicate and sensitive organization, are able to perceive and rightly estimate the nature of individuals, by being brought in contact with any substance which has passed under their hands or any other thing upon which they may have bestowed a thought, and this too, after the intervention of long periods of time. The exercise of this gift, has been denominated *psychometry*, or soul measure, and the adepts of it are multiplying almost daily. This truth so well established explains the whole mystery of the mind cure, for there is great potency for good or evil, in these spiritual or soul eminations, hence, when this mysterious force is united in a number of individuals of comparatively pure and blameless lives, and concentrated upon a diseased person whose mind is receptive to its influences, great good to the sufferer may, and is likely to result from it, by arousing his dormant energies, restoring his lost hope and leading his mind unto a belief in his ultimate restoration to health, which if accomplished, the battle is more than half won, for nature thus assisted and encouraged, immediately sets about restoring the disordered elements to a normal condition. Neither the prayers of the zealots nor the faith of the afflicted, except so far as they serve to create harmony on the one part, and receptively on the other, have anything to do with it.

It is nature's self that accomplishes the desired end and wholly through natural means irrespective of creed add ceremonies and forms of belief.

> "All nature is but art unknown to thee,
> All chance, direction, which thou canst not see."

The laws of being, are universal and everywhere and at all periods the same : the same for Pagans as for Christians, alike for the African in his rude hut, and the European in his luxurious palace. The effort of our lives should be acquire a knowledge of these laws and bring ourselves into harmony with them. All counter efforts inevitably result in failure.

